# Attention, working memory and executive functions in patients with internet addiction disorder 

**Published:** 2019-08

**Authors:** Ali Reza Shafiee-Kandjani, Zahra Mohammadzadeh, Shahrokh Amiri, Arfaie Asghar, Parvin Sarbakhsh, Safikhanlou Salman

**Affiliations:** ^*a*^Road Traffic Injury Research Center, Department of Psychiatry, Tabriz University of Medical Sciences, Tabriz, Iran.; ^*b*^Psychiatry Resident, Research Center of Psychiatry and Behavioral Sciences, Tabriz University of Medical Sciences, Tabriz, Iran.; ^*c*^Research Center of Psychiatry and Behavioral Sciences, Tabriz University of Medical Sciences, Tabriz, Iran.; ^*d*^Department of Epidemiology and Biostatistics, School of Health. Tabriz University of Medical Sciences, Tabriz, Iran.; ^*e*^Clinical Psychologist, Research Center of Psychiatry and Behavioral Sciences, Tabriz University of Medical Sciences, Tabriz, Iran.

## Abstract

**Background:**

Attention, working memory and executive functions are high cortical activities which can be impaired in patients with internet addiction disorder (IAD). Many studies have shown a considerable cognitive deficit compared with healthy individuals. The aim of the study was to determine the possible deficits in the afflicted patients in an Iranian population.

**Methods:**

In this descriptive- analytic study, 30 patients with internet addiction disorder were recruited compared with 30 healthy counterparts through semi-structured interview (SCID), Internet Addiction Test (IAT) and General Health Questionnaire (GHQ). The drop-out rates were 10 and 13 percent for IAD and control group, respectively. Cognitive functions were assessed by Persian Paper and Pencil Cognitive Assessment Package (PCAP).Data were analyzed through SPSS-23 by Analysis of variance and chi- square. P- Values less than 0.05 were considered statistically significant.

**Results:**

Stroop test and Verbal fluency test showed no significant difference in attention and executive functions between the groups (p>0.05) but Backward Digit Span Test (BDST) and Letter Number Sequencing Test (LNST) revealed a significant difference (p< 0.001).

**Conclusions:**

The results showed that patients with IAD were suffering from working memory impairments which may impact on educational functioning especially among students.

**Keywords:**

Attention, Working memory, Internet addiction

